# Do transgenesis and marker-assisted backcross breeding produce substantially equivalent plants? - A comparative study of transgenic and backcross rice carrying bacterial blight resistant gene Xa21

**DOI:** 10.1186/1471-2164-14-738

**Published:** 2013-10-29

**Authors:** Lifen Gao, Yinghao Cao, Zhihui Xia, Guanghuai Jiang, Guozhen Liu, Weixiong Zhang, Wenxue Zhai

**Affiliations:** 1 Institute of Genetics and Developmental Biology, Chinese Academy of Sciences, Beijing 100101, China; 2 Institute for Systems Biology, Jianghan University, Wuhan, Hubei 430056, China; 3 College of Life Sciences, Hebei Agricultural University, Baoding, Hebei 071001, China; 4 Department of Computer Science and Engineering, Washington University in St. Louis, St. Louis, MO 63130, USA

**Keywords:** Transgenesis, Marker-assisted backcrossing, *Substantial equivalence*, Transcriptome profile, *Xa21*

## Abstract

**Background:**

The potential impact of genetically modified (GM) plants on human health has attracted much attention worldwide, and the issue remains controversial. This is in sharp contrast to the broad acceptance of plants produced by breeding through Marker Assisted Backcrossing (MAB).

**Results:**

Focusing on transcriptome variation and perturbation to signaling pathways, we assessed the molecular and biological aspects of substantial equivalence, a general principle for food safety endorsed by the Food and Agricultural Organization and the World Health Organization, between a transgenic crop and a plant from MAB breeding. We compared a transgenic rice line (DXT) and a MAB rice line (DXB), both of which contain the gene *Xa21* providing resistance to bacterial leaf blight. By using Next-Generation sequencing data of DXT, DXB and their parental line (D62B), we compared the transcriptome variation of DXT and DXB. Remarkably, DXT had 43% fewer differentially expressed genes (DEGs) than DXB. The genes exclusively expressed in DXT and in DXB have pathogen and stress defense functions. Functional categories of DEGs in DXT were comparable to that in DXB, and seven of the eleven pathways significantly affected by transgenesis were also perturbed by MAB breeding.

**Conclusions:**

These results indicated that the transgenic rice and rice from MAB breeding are substantial equivalent at the transcriptome level, and paved a way for further study of transgenic rice, e.g., understanding the chemical and nutritional properties of the DEGs identified in the current study.

## Background

The primary objective of breeding in agriculture is to develop plants of desired genotypes or traits, such as high yields and resistance to adverse environmental impact. Marker-assisted backcrossing (MAB) and transgenesis (aka genetic modification or GM) are two widely adopted plant breeding techniques. As a conventional technique, MAB breeding has been used to develop new crop cultivars of, e.g., baley, maize and rice [1-4]. The basis of MAB breeding [5] is to transfer a specific allele at the target locus in a donor line to a recipient line while selecting against donor introgression across the rest of the genome. In most cases, the recipient line used for backcrossing has a large number of favorable attributes but is deficient in a few characteristics. Since MAB breeding to certain degree mimics or replicates natural selection, novel cultivars produced through MAB breeding have been regarded as genetically safe. However, MAB breeding is laborious, requiring several backcrossings and a large number of individual plant screenings, typically on the order of thousands. It typically takes a great deal of luck to produce a product of a desirable trait.

Transgenesis is an effective means for improving crop genetic makeup for deriving favorable traits. Breeding by transgenesis has several advantages over breeding by MAB. Transgenesis is a direct means for introducing a gene or genotype to a genome in order to produce a target trait. As a result, the potential to produce plants with favorable traits increases dramatically. For example, by transfering a gene that encodes a plastidial adenyalte kinase into a potato variety, the transgenic potato displays wild-type growth and developmental phenotype but also has an increased yield and starch concentration [6]. By introducing the hybrid cellulase gene *cel-hyb1* into a spring barley variety through Agrobacterium-mediated transformation, the selected marker-free transgenic barley produces a high level of cellulase (1,4-β-glucanase) in developing grains, suggesting that the transgenic barley has the potential for producing a large quantity of cellulase for commercial use [7]. The nutritional value of Golden Rice is improved with increased pro-vitamin A content by introducing genes encoding phytoene synthase (*psy*) in combination with the Erwinia uredovora carotene desaturase (*crtI*) into rice [8]. Through an Agrobacterium-mediated genetic transformation system, *Xa21*, a rice bacterial blight resistance gene, has been introduced into five Chinese rice varieties and as a result, the transgenic rice plants exhibit a high resistance to bacterial blight [9].

While transgenesis offers immense opportunities to curtail the severe threat of food shortage the expanding world population is facing, there are considerable public concerns over the use of transgenesis for crop improvement. Indeed, it remains extremely controversial whether or not transgenic crops have an adverse impact on human health. At the center of this controversy is the issue of whether or not insertion of a transgene into the host plant genome or manipulation of an allele in the host genome may affect the expression of other genes and ultimately lead to unintended phenotypes. Unfortunately, it is technically challenging to address this issue because accurate prediction of phenotypes based on genotypic variation and/or gene expression alteration remains a research topic.

Due to the difficulty, efforts of evaluating the safety of transgenic crops has been geared toward assessing the substantial equivalence between transgenic plants and wildtype or conventionally bred plants, like plants from MAB breeding [10]. *Substantial equivalence* has been introduced as a standard by the Organization for Economic Cooperation and Development (OECD) and has been endorsed by the Food and Agriculture Organization of the United Nations/World Health Organization (FAO/WHO) [11]. However, the standard is based on comparative analysis and offers only a general principle. No specific molecular, biological, chemical or nutritional basis has been established to precisely specify the degree of substantial equivalence [12,13]. It thus leaves widely open the study of various aspects of equivalence, ranging from molecular, biological, and chemical to nutritional equivalence, between a transgenic plant and a wildtype or plant produced by MAB breeding. Nevertheless, it has been agreed that to be considered substantially equivalent, the characteristics of a transgenic plant must be within a natural range of variation [14], a guideline we follow in our study.

Rice is an essential staple crop for the world population and a model plant for basic and applied research. Rice bacterial leaf blight (BLB), caused by bacteria *Xanthomonas oryzae* pv. *oryzae* (*Xoo*), is one of the most devastating rice diseases throughout the world. Utilization of BLB resistant genes in breeding is the most effective and economical strategy for controlling BLB [15]. *Xa21*, the first-cloned BLB resistant gene from *Oryza longistaminata*, has received much attention because of its broad spectrum of resistance to BLB [16]. The gene has also been widely used in BLB resistance breeding through both the transgenic and MAB strategies [17-21]. Therefore, transgenic rice and MAB rice carrying *Xa21* offer an excellent opportunity to assess the possible substantial equivalence of transgenic and MAB rice as well as rice in natural environments.

In order to pave the way for future studies of the safety of transgenic crops, we focused on the molecular and biological aspects of substantial equivalence of transgenic rice. We adopted a systems-biology perspective and examined the transcriptome variation of transgenic rice. Specifically, we incorporated *Xa21* into the three-line maintainer line D62B through transgenesis and MAB breeding. Adopting Next Generation (Next-Gen) sequencing, we profiled the transcriptomes of four rice plants: the *Xa21* transgenic line (named as DXT), the *Xa21* MAB breeding line (named as DXB), the untransformed recipient D62B, and another rice variety MH86 (restorer line). We then analyzed transcriptome variation of the two rice plants carrying *Xa21* in reference to that of D62B and transcriptome change between D62B and MH86. This transcriptome analysis was further enhanced by a pathway analysis to understand the pathways that might be disturbed in the two rice plants carrying *Xa21*.

## Results

### A system for comparative study of transgenic and MAB rice

In order to compare transgenic and MAB plants, transgenic and MAB rice plants carrying *Xa21* using the parental line D62B were constructed. To generate the transgenic rice, *Xa21* was introduced into D62B through *Agrobacterium*-mediated transgenesis. The transgenic rice plants were selected from the T_0_, T_1_ and T_2_ generations by molecular and resistance analysis. The homozygous, single copy, and marker-free transgenic line, or DXT for short, was obtained from the T_2_ generation. To confirm the results, Southern hybridization analysis was performed using the restriction endonuclease *Pvu*II to digest genomic DNA from the transgenic and parental plants. The details for developing the transgenic rice was described in our early report [22]. The sample used for analysis was the T_9_ generation of DXT with stable agronomic traits.

*Xa21* was introgressed into the parental line D62B to produce the MAB breeding line using IRBB21 as the donor. IRBB21 was bred by transfering *Xa21* into IR24 through backcrossing [23]. Six backcrossing generations were made because it is usually necessary to take a minimum of six backcrossing generations in order to recover the phenotype of recurrent parent lines and eliminate donor chromosome fragments linked to the target gene [5]. A backcrossed line with homozygous *Xa21* and similar phenotype with the recipient D62B was obtained in BC_6_F_2_ generation and named DXB.

In order to facilitate direct in-field screening and molecular analysis of transgenic and MAB plants that showed consistent agronomic traits similar to that of their parental line, the transgenic line (DXT), the MAB line (DXB) and their parental line (D62B) were grown in the same fields in the breeding process. D62B can thus serve as an ideal control for the comparison of DXT and DXB. In order to introduce a reference to natural variation, another rice varieties MH86, an *indica* restorer line in the three-line breeding system was also included in the profiling experiments. Since rice carrying *Xa21* confers robust resistance to most strains of *Xoo* at adult stages, the RNA samples were extracted from adult leaves of the four rice lines for transcriptome profiling.

### The transgenic rice and MAB rice were phenotypically similar

**Figure 1:**
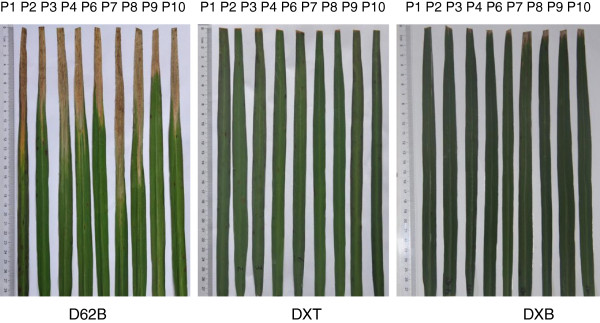
**Bacterial blight resistance spectrum analysis of D62B, DXT and DXB.** Nine *Xoo* strains from Philippine (P1-P10) were used to innoculate the rice leaves in the high tillering stage. The shorter the yellow portion on a leaf is, the higher plant resistance to *Xoo* infection. DXT: transgenic line with *Xa21*; DXB: MAB line with *Xa21*; D62B: the common parental line of DXT and DXB.

The morphological characteristics of DXB, DXT, D62B, and MH86 were examined in the rice fields. DXT and DXB were morphologically similar to their parental line under visual inspection. The major agronomic traits of DXT and DXB, listed in Table 1, were also scored in the fields. The results showed that the main agronomic traits of DXT and DXB were consistent with or comparable to that of D62B, except with respect to the focal trait of BLB resistance. Both DXT and DXB were highly resistant to nine Philippine races of *Xoo* but D62B was susecptible to the all *Xoo* races (Figure 1). Highly phenotypic similarity among DXT, DXB, and D62B suggested that both transgenic and MAB breeding strategies had very little impact on the morphological characteristics and main agronomic traits of D62B other than the expected BLB resistance.

**Table 1:** Major agronomic traits of four rice plants studied

**Sample**	**Lesion length (cm)**	**Plant height (cm)**^**a**^	**Tillers**	**Panicle length (cm)**^**b**^	**Seed ratio (%)**	**1000-grains weight (g)**
**D62B**	10.0±2.7	88.4±4.3	11±3	23.9±1.2	94±3.0	25.8±2.8
**DXT**	0.4±0.3^#^	86.3±2.0	10±1	24.2±0.9	93±4.7	25.8±2.2
**DXB**	1.2±0.4^#^	85.5±3.4	12±2	23.8±1.1	93±2.9	26.0±1.7
**MH86**	11.3±1.9	113.1±2.8*	10±2	27.0±1.7*	80±5.8*	27.1±1.0

Data included are averages of at least 5 individual plants from which 3 infected leaves (a) or panicles (b) were scored. * represents a significant levels of *p* < 0.01 beween D62B and MH86 (*t*-test). # represents the significant levels of *p* <0.01 in DXT and DXB with D62B as the control.D62B: the recipient control; DXT: transgenic line with *Xa21*; DXB: MAB line with *Xa21*; MH86: another rice variety.

In contrast, there were evident morphological differences between the two distinct rice varieties D62B and MH86. The sheath and blade of MH86 were light green but that of D62B were reddish dark green. Moreover, D62B showed a narrower blade, thinner and shorter stem, and a shorter growth period than MH86.

### Transcriptome profiling using Next-Gen sequencing

In order to investigate transcriptome variations of the transgenic and MAB rice, digital gene expression profiles of DXT, DXB, D62B and MH86 were obtained using Next-Gen sequencing. The 5.8 to 6.1 million raw sequencing reads (Table 2) for the samples have been deposited into NCBI/GEO (accession number SRA061839). Preprocessing of these raw data (see Methods) gave rise to 5.7 to 6.0 million qualified reads for down-stream analysis. The qualified reads were mapped to the cDNA sequences of *Oryza Sativa Nipponbare* with a stringent criterion, giving 12.1 to 13.3 thousand unigenes per sample (Table 2). A statistical analysis (see Methods) of all the genes mapped in the four samples detected 15,268 unigenes, among which 10,362 were common to all four rice plants. Based on their abundance, measured in reads per kilobase of exon model per million mapped reads (RPKM), 508 (4.9%), 3,786 (36.5%) and 6,068 (58.6%) of the 10,362 common genes were expressed at high (RPKM >100), medium (10 < RPKM ≤100) and low (RPKM ≤10) levels, respectively. The results indicated that the majority of the expressed genes had low or median abundance.

**Table 2:** Statistics of RNA sequencing data, generated by Illumina sequencing, on four rice plants

	**D62B**	**DXT**	**DXB**	**MH86**
Raw reads	5,801,661	6,161,959	6,101,257	6,104,443
Qualified reads	5,677,504	6,026,127	5,962,165	5,954,422
Unambiguous tag mapping to Gene	2,330,333	2,724,696	2,549,799	2,665,554
Unambiguous tag-mapped Genes	12,048	12,686	12,526	13,349
Unambiguous tag/gene	193.4	214.8	203.6	199.7

D62B: the recipient control; DXT: transgenic line with *Xa21*; DXB: MAB line with *Xa21*; MH86: another rice variety.

### Transgenesis induced smaller transcriptome variation than MAB breeding

A screening of differentially expressed genes (DEGs) revealed 984 DEGs (739 up-regulated and 245 down-regulated) in DXT vs. D62B, 1413 DEGs (938 up- and 475 down-regulated) in DXB vs. D62B, and 2599 DEGs (2109 up- and 490 down-regulated) in MH86 vs. D62B (Additional file 1: Table S1). Eight up-regulated and two down-regulated genes in DXT were further analyzed by qRT-PCR (Additional file 2: Figure S1). Eight of these ten genes profiled exhibited expression consistent with the sequencing data, confirming the results from Next-Gen sequencing; seven of the eight up-regulated genes were indeed significantly up-regulated and one of the two down-regulated gene was significantly down-regulated in DXT (Additional file 2: Figure S1). The discrepancy between the results of deep sequencing and qRT-PCR on two genes assayed may be due to the technical difference between the two techniques [24,25].

**Figure 2:**
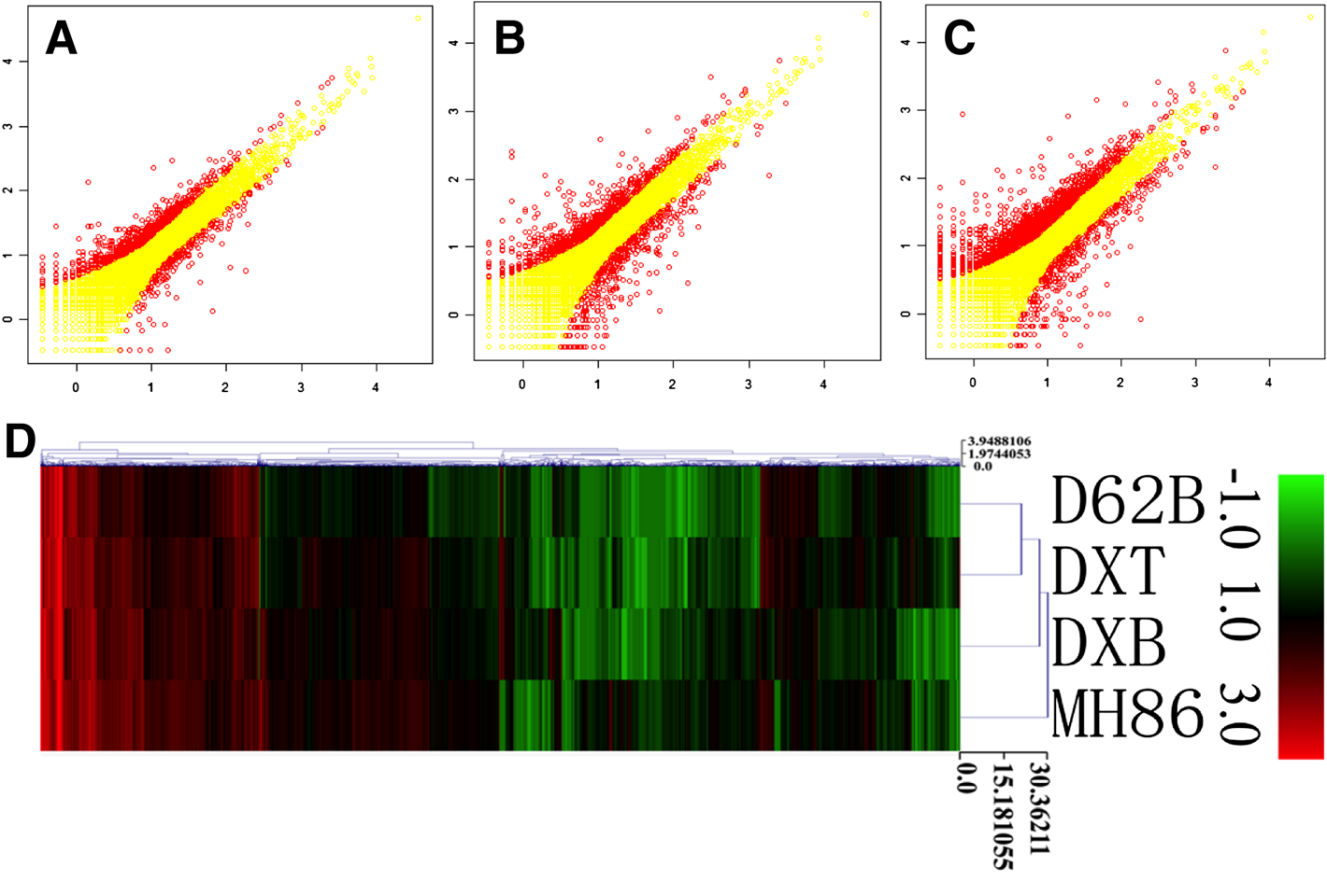
**Transcriptome variations and expression relationships among four rice plants studied.** Scatter plotes of transcriptome comparison of **(A)** DXT vs. D62B, **(B)** DXB vs. D62B, and **(C)** MH86 vs. D62B, where the horizontal and vertial axes represent the digital gene expression abundance after log10 transformation, and dots in red represent differentially expressed genes. **(D)** Clustering results on the differentially expressed genes of DXT, DXB and MH86 with respect to D62B. Each column in the figure refers to a gene. Digital expression abundance, after log10 transformation, is used in the plot. DXT: transgenic line with *Xa21*; DXB: MAB line with *Xa21*; D62B: the common parental line of DXT and DXB; MH86 :another rice variety.

Remarkably, the transcriptome variations between the two rice varieties D62B and MH86, measured by the number of DEGs between the two, was the largest, while the transcriptome variations between DXT and DXB was the smallest among the comparisons. More importantly, transgenesis of *Xa21* induced 43.6% less transcriptome variation than MAB breeding. The differences among such transcriptome variations were displayed in Figures 2A to 2C. Overall, the results showed that the transcriptome change due to the introduction of *Xa21* was significantly smaller than that by MAB breeding and the variation between the transcriptomes of two rice varieties D62B and MH86.

Furthermore, a clustering analysis of DEGs (see Methods) showed that the rice plants with close genetic background tended to group together (Figure 2D). As expected, DXT and DXB were closer to their parental line D62B than they were to MH86. Despite the difference in the two breeding strategies, the transgenic line DXT was more closely related to D62B than the MAB line DXB (Figure 2D). These results revealed that transgenesis and MAB breeding did not alter the transcriptome more significantly than another natural rice variety (i.e., MH86). As far as transcriptome variation is concerned, these results suggest that the transgenic rice is closer to its parental line than the MAB rice is to the same parental line.

### Transgenesis had less impact on molecular and cellular functions than MAB breeding

**Figure 3:**
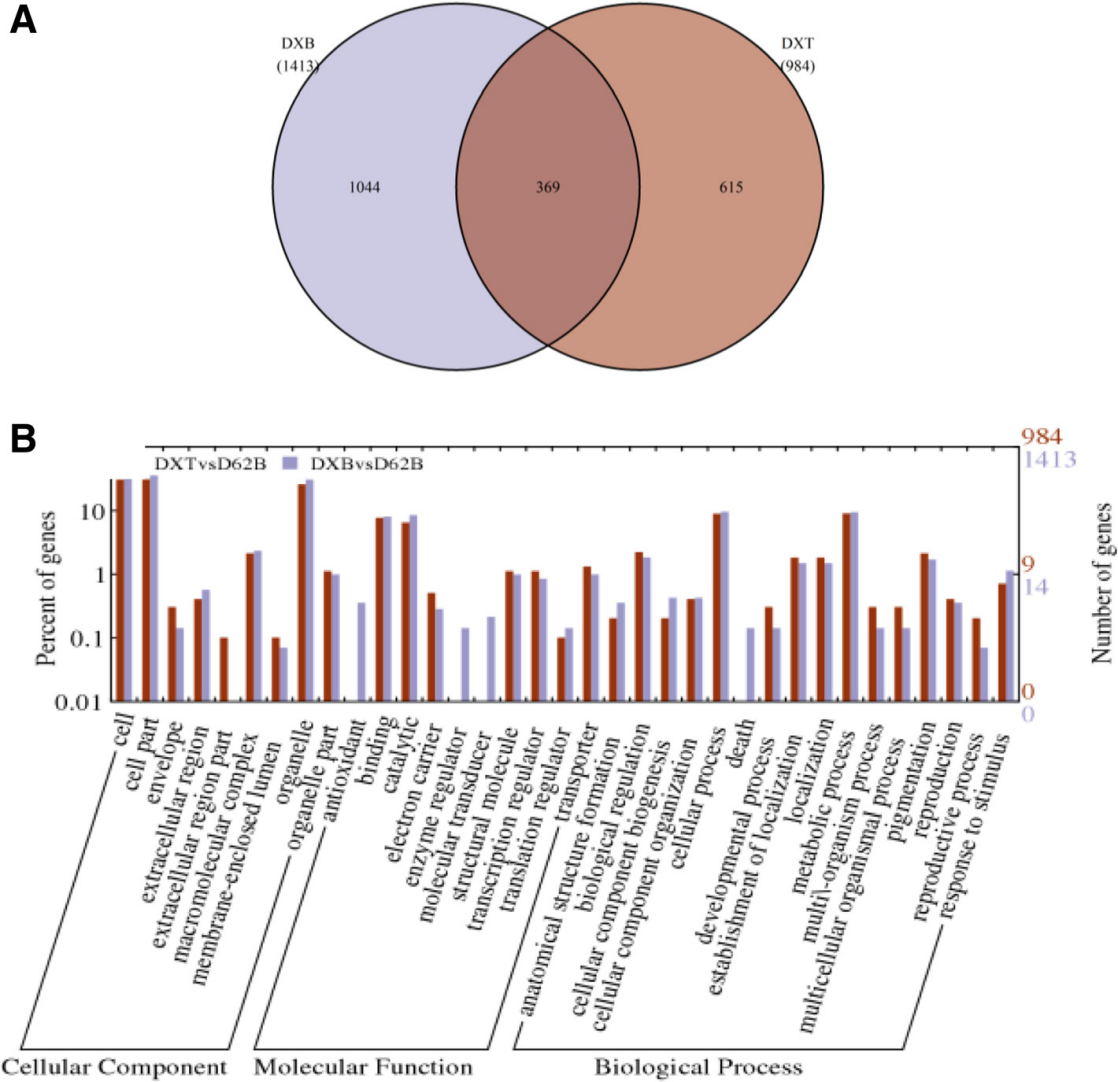
**Comparative analysis of the DEGs in DXT and DXB with respect to D62B. (A)** Venn diagram for the numbers of DEGs in DXT and DXB. **(B)** GO functional categories of the DEGs in DXT and DXB. The y-axis on the right indicates the number of genes in a category, and the y-axis on the left is the percentage of genes to be analyzed in a category. DXT: transgenic line with *Xa21*; DXB: MAB line with *Xa21*; D62B: the common parental line of DXT and DXB.

In order to appreciate the possible consequences of introducing *Xa21* into D62B by the two breeding strategies, GO functional analysis was performed on the DEGs between DXT and D62B and between DXB and D62B (Figure 3). In total, 31 and 35 functional categories were enriched among the DEGs between DXT and D62B and between DXB and D62B, respectively. Surprisingly, 30 of these enriched functional categories were common (Figure 3B), indicating that most of the DEGs in DXT and DXB have the same or similar functions. The single functional category specific to the DEGs in DXT is the “extracellular region part”, and the four categories unique to the DEGs in DXB include “antioxidant”, “enzyme regulator”, “molecular transducer” and “death” (Figure 3B). Although it is unclear what phenotypic consequence, if any, that these DEGs may lead to, it is evident that the transcriptome variation caused by the DEGs in DXT imposed less cellular perturbation than that by the DEGs in DXB. In other words, the transgenesis of *Xa21* induced less disturbance to the molecular and cellular machineries than the MAB breeding did.

**Figure 4:**
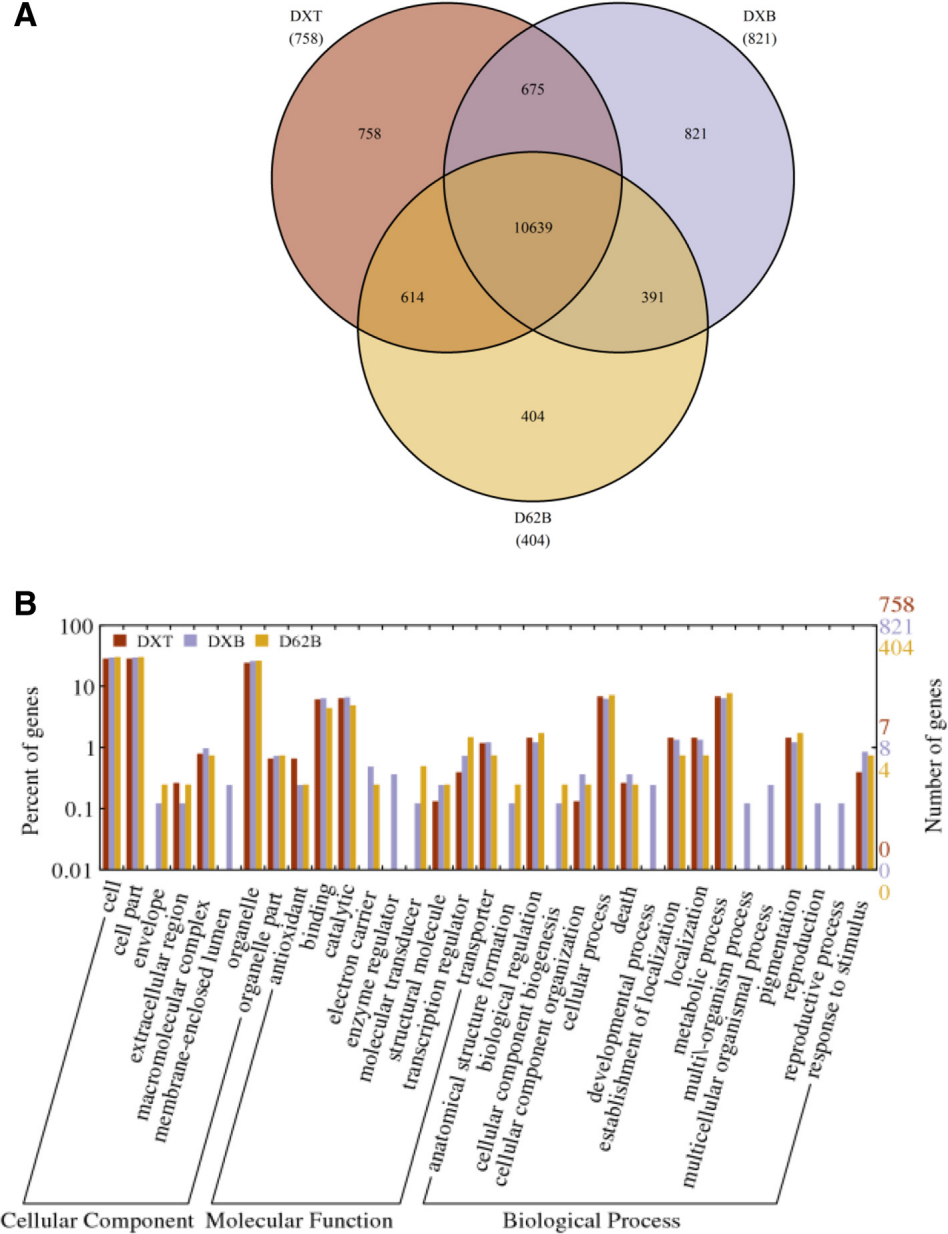
**Comparative analysis of the genes exclusively expressed in transgenic line DXT, MAB breeding line DXB and their parental line D62B. (A)** Venn diagram for the numbers of genes exclusively expressed in DXT, DXB and D62B. **(B)** GO functional categories of the genes in DXT and DXB. The y-axis on the right indicates the number of genes in a category, and the y-axis on the left is the percentage of genes to be analyzed in a category. DXT: transgenic line with *Xa21*; DXB: MAB line with *Xa21*; D62B: the common parental line of DXT and DXB.

To further understand the effects of the two different breeding strategies, we analyzed the genes expressed exclusively in DXT and exclusively in DXB, along with the ones expressed exclusively in D62B. We identified 758, 821 and 404 genes that were exclusively expressed in DXT, DXB and D62B, respectively (Figure 4A). The DXT-, DXB- and D62B-specific genes were enriched in 21, 33 and 26 GO functional categories, respectively (Figure 4B). Interestingly, the 21 categories enriched in the DXT-specific genes were also enriched in the DXB- and D62B-specific genes, and the 26 categories enriched in the D62B-specific genes were enriched in the DXB-specific genes as well (Figure 4B). This result indicated that although there were a large number of genes expressed exclusively in these plants, they carried the same or similar molecular and/or cellular functions, reflecting the functional elasticity and robustness of these genes. On the other hand, comparing with the genes expressed in the parental line, the genes expressed exclusively in DXT lost functions in 5 categories, while the genes expressed exclusively in DXB gained additional functions in 7 categories. It is possible that these lost and gained functions may be compensated for by the genes commonly expressed in these three rice plants. Nevertheless, this plant-specific gene function analysis also indicated that the perturbation to the molecular and cellular functions induced by transgenesis has a smaller or compatible scale than by MAB breeding.

### Genes exclusively expressed in transgenic rice functioned in pathogen defense

Among the DXT-specific genes, 52 encoded transposons and retrotransposons, supporting the notion that mobile elements are typically activated during genetic transformation [26]. In addition, 40 DXT-specific genes encoded protein kinases, receptor-like protein kinases (RLKs), or OsWAK receptor-like protein kinases that have been implicated in stress/defense signaling-signal perception and signal transduction, which are consistent with the existing results that show that protein phosphorylation and dephosphorylation play an important role in *Xa21* induced gene regulation in response to pathogen invasion [27-32]. The remaining DXT-specific genes included 14 genes encoding transporter (including potassium, sulfate, metal cation, ABC, and ctr copper transportor), 16 genes encoding putative transcription factors (including 6 AP2 domain containing proteins, 1 ethylene-responsive transcription factor (ERF), and 9 MYB family transcription factors), 8 DXT-specific expressed genes encoding resistance or resistance-like proteins (including 5 NBS-LRR type of proteins such as LR10 [33], RPM1 [34] and I2GA-SH194-2 [35]) and 2 genes encoding DNA methylation related proteins. DNA methylation has been reported to affect rice resistance response [36-38]. The AP2/ERF family is a large family of plant specific transcription factors that share a well-conserved DNA-binding domain that has been reported to activate the expression of abiotic stress-responsive genes via specific binding to the dehydration-responsive element/C-repeat (DRE/CRT) cis-acting element in their promoters [39]. The MYB family has key transcription factors for controlling plant development and response to biotic and abiotic stresses [40]. Taken together, these results strongly suggested that transgenesis of *Xa21* affected mainly those genes that were involved in pathways related to stress response.

Compatible observations were also made on the genes expressed exclusively in DXB, i.e., these genes had the same or similar functions as those exclusively expressed in DXT. Among the DXB-specific genes, 58 encoded transposons and retrotransposons and 24 encoded protein kinases, receptor-like protein kinases (RLKs) or OsWAK receptor-like protein kinases. The remaining DXB-specific genes included 10 genes that encode transporters, 10 that encode putative transcription factors (including 3 MYB domain containing proteins), 13 that encode resistance or resistance-like proteins (including 7 NBS-LRR type of proteins), and 3 that encode DNA methylation-related proteins.

### Most pathways perturbed by transgenesis were also disturbed by MAB breeding

Our final step of the comparative study of transcriptome variation was a pathways analysis on the DEGs in DXT and the DEGs in DXB. To this end, the DEGs in these two plants were mapped to the pathways in the Kyoto Encyclopaedia of Genes and Genomes (KEGG) database and searched for significantly enriched KEGG pathways that were potentially affected by gene expression variation [41]. Among the DEGs in DXT and DXB, 476 and 700 were annotated on KEGG pathways, respectively. In comparison, more DEGs, i.e., 1331, between MH86 and D62B were mapped to KEGG pathways. With a statistical significance of *p*≤0.05; 11, 16, and 20 signaling pathways were abundant among the DEGs of DXT, DXB and MH86, respectively (Table 3, Additional file 1: Table S2). Seven of the 11 significantly enriched pathways in the transgenic rice were also significantly affected in the MAB rice. The rest four pathways (including Carotenoid biosynthesis, Regulation of autophagy, Arachidonic acid metabolism and Anthocyanin biosynthesis) were also detected in the MAB rice but at a less significant level above the cutoff threshold (Additional file 1: Table S3). Specifically, “Carotenoid biosynthesis”, “Regulation of autophagy”, “Arachidonic acid metabolism”, and “Anthocyanin biosynthesis” had *p*-values of 0.22, 0.88, 0.06, and 0.67 respectively in the MAB rice.

**Table 3:** Pathways enriched in differentially expressed genes in the transgenic line DXT, MAB breeding line DXB and a rice variety MH86 with respect to recipient line D62B

**Pathways**	**DXT**	**DXB**	**MH86**
Ribosome	√	√	√
Flavonoid biosynthesis	√	√	√
Vitamin B6 metabolism	√	√	√
Biosynthesis of phenylpropanoids	√	√	
Benzoxazinoid biosynthesis	√	√	
Flavone and flavonol biosynthesis	√	√	
Oxidative phosphorylation	√	√	
Carotenoid biosynthesis	√		
Regulation of autophagy	√		
Arachidonic acid metabolism	√		
Anthocyanin biosynthesis	√		
Zeatin biosynthesis		√	√
ABC transporters		√	√
Inositol phosphate metabolism		√	√
Phenylalanine metabolism		√	
Glycine, serine and threonine metabolism		√	
Phagosome		√	
Biosynthesis of secondary metabolites		√	
Circadian rhythm - plant		√	
Sulfur metabolism		√	
Metabolism of xenobiotics by cytochrome P450			√
Spliceosome			√
Endocytosis			√
Biosynthesis of plant hormones			√
Biosynthesis of unsaturated fatty acids			√
Glutathione metabolism			√
Linoleic acid metabolism			√
Biosynthesis of terpenoids and steroids			√
alpha-Linolenic acid metabolism			√
Phosphatidylinositol signaling system			√
Limonene and pinene degradation			√
Non-homologous end-joining			√
Biosynthesis of alkaloids derived from ornithine, lysine and nicotinic acid			√
Glycosylphosphatidylinositol(GPI)-anchor biosynthesis			√

Listed are significantly enriched KEGG terms with cutoff of *p* ≤ 0.05. “√” means significantly affected pathway. Note that the four pathways enriched in DXT but not listed under DXB in the table were also enriched in DXB at a less significant level (with *p*-values from 0.06 to 0.88).

## Discussion

MAB breeding is a well accepted breeding technology for production of safe crops. Comparing crops produced from transgenesis and MAB breeding thus offers a viable approach to assessing the potential impact that transgenic crops might have on human health. As a general principle, substantial equivalence [11] can be followed to assess the safety of transgenic crops. Using this principle as a guideline and yardstick, various aspects of substantial equivalence, e.g., molecular, biological, chemical and nutritional equivalence, can be examined. Taking a systems-biology perspective, we focused on molecular and biological equivalences between transgenic crops and plants from MAB breeding in the current study. This was done by contrasting transcriptome variations of a transgenic rice and a rice by MAB breeding. As a caveat, any results from such a study should not be interpreted to directly address the safety of transgenic plants. But rather, in addition to gaining insights, at a genome scale, into the biological processes and pathways that might be perturbed by transgenesis, such results can be used to pave the way for further study of chemical and nutritional equivalence.

Adopting the above view point and taking *Xa21*, the most widely used resistance gene to rice bacterial leaf blight, as the target gene, we studied the phenotype and transcriptome differences between the transgenic and MAB rice carrying *Xa21*. Included in our expriment system were a transgenic rice (i.e., DXT) and a MAB rice (i.e., DXB) that were developed from the same parental line D62B. DXT was identical to D62B except the key *Xa21* gene. The genetic makeup of DXB, through careful selection over six generations of backcrossing to D62B, was almost identical to that of D62B. Therefore, with exception of the specific breeding techniques used, DXT and DXB carried nearly identical genetic materials, making them ideal for the comparison of the two breeding techniques. A critical component of our experiments and analysis was another natural rice variety, MH86, which does not carry *Xa21*. Here, MH86 played the role of a“control”, when compared with D62B, to reveal the natural range of transcriptome variation between the two natural rice plants.

The comparison of transgenic and MAB rice was done at two levels. At the physiological level, we confirmed through extensive, although laborious, in-field screening of various morphological properties to ensure that DXT and DXB were morphologically equivalent or similar. Due to genetic diversity, the morphological difference between natural varieties are typically greater than that of transgenesis, as we saw in our comparison between MH86 vs. D62B and DXT vs. D62B. At the molecular and cellular level, we carefully studied transcriptome variations as well as function and pathway alterations in DXT and DXB. The scale of transcriptome variations caused by transgenesis was smaller than that by MAB breeding. More importantly, the molecular and cellular functions that may be affected by transgenesis were also affected by MAB breeding, and the majority of pathways perturbed in the transgenic rice were also distorted in the MAB rice. In stark contrast, transcriptome and function variations between MH86 and D62B, which can be regarded as natural variations, were substantially larger than that between DXT and D62B. In short, the variations caused by transgenesis were smaller than that of MAB breeding and were within the natural range of variations.

The results from the current study were in agreement with that in the literature on transgenic wheat and maize. For example, Gregersen *et al*. compare the gene expression profiles of wildtype and transgenic wheat expressing an *aspergillus fumigatus* phytase [42]. The results show that the expression profiles of the two plants are not significantly different. A similar study of transgenic wheat, which carries additional High Molecular Weight subunit genes, reaches the same conclusion that transgenesis has less impact on the transcriptome of wheat grain than conventional breeding [10]. Further, Coll *et al.* uses microarrays to compare the expression profiles of commercial maize variety MON810 and near-isogenic varieties in leaves *in vitro* and also field cultured plants of AristisBt/Aristis and PR33P67/PR33P66 [43,44]. The target gene is *Bt*, a gene non-native to maize encoding insecticidal crystal protein of the soil *bacterium Bacillus thuringiensis* (*B.t.*). The results show that gene expression profiles of MON810 and comparable non-GM maize varieties are more similar to that of conventional lines and natural variation.

On the other hand, the experiment designs of previous studies are far from perfect. In [10], comparative analysis of the transcriptomes of three wheat lines, including a parental line, a conventional breeding line and a transgenic line, reaches the conclusion that transgenesis has less impact on the transcriptome of wheat grain than conventional breeding. However, several additional genes or sequences, including the marker gene (*Bar* gene), the reporter gene (*uidA* gene), and sequences derived from the bacterial plasmid, are present in the transgenic line. The transgenic line, referred to as a “clean fragment” line, still contains the *bar* gene. In fact, selective markers, such as *bar,* are the main concern of biosafety of transgenic crops which induce specific pleiotropic effects [45]. In the studies reported in [42-44], foreign transgenes are used. The current study overcame these issues. First, the target gene *Xa21* was cloned from *Oryza longistaminata*, which is native to rice. Second, other than *Xa21* sequence, DXT did not carry any additional sequences such as that for marker genes and reporter genes. Third, unlike the previous studies where a target gene is under the control of a foreign promoter [42,46], in the current study *Xa21* in DXT carried its own promotor. As a result of these factors, the results from our analysis were specific to the functions of *Xa21* rather than some artifacts of genes foreign to rice.

More importantly, our analysis expanded beyond transcriptome profiling to include analyses of gene functions and signaling pathways that might be altered by the introduction of *Xa21*. Our results showed that most of the molecular and cellular functions affected by transgenesis were influenced by MAB and functional categories that affected by MAB were more than those that affected by transgenesis. Analyzing the pathways perturbed by transgenesis and MAB showed that majority pathways altered in transgenic rice were also distorted in MAB rice. The bigger difference between the transcriptome variations of DXB and D62B could be attributed to two factors. Firstly, the transgenic rice analyzed in our study was carefully selected to have favorable properties for the purpose of the study. In particular, DXT was a single-copy, marker-free, and homozygous *Xa21* transgenic line and also had consistent agronomic traits similar to that of the parental line D62B. The genetic makeup of DXT was identical to D62B with the exception of the *Xa21* gene. Secondly, although six backcrosses have been used to eliminate linkage drag, a few unwanted donor chromosomal segments could still have been retained in DXB when *Xa21* was introgressed into D62B. Therefore, the transcriptome of DXT was closer to that of D62B than DXB to D62B.

Thanks to Next-Gen sequencing, the gene profiling experiments of our study were genome wide and provided a high resolution [47]. In contrast, the previous studies focus on a limited number of genes using microarray profiling which is less accurate and restricted to annotated genes [10,42-44,46,48]. Such a large scale functional analysis performed in the current study has never been attempted before and the functional analyses provided deep insight into the functional equivalence between the transgenesis and MAB breeding.

## Conclusions

MAB breeding and transgenesis are two most popular breeding techniques for producing plants of favorable traits. As a newer biotechnology, transgenesis has made visible contributions to increase yield of staple crops. While plants produced by MAB breeding have already been widely accepted, transgenic plants are facing the challenge regarding safety on human health. In the current study, these two distinct breeding techniques was closely compared. The analysis focused on transcriptome variations in rice plants generated from these two breeding techniques and on that of two natural rice plants as a baseline of the comparison. The study combined careful assessment of agronomic traits, transcriptome profiling by Next-Gen sequencing, and functional and pathway analyses. Two important conclusions can be drawn from the results. First, transcriptome variation caused by transgenesis is significantly smaller than that by MAB breeding and is within the range of natural variation. Second, the functional categories of differentially expressed genes due to these two breeding techniques and the pathways perturbed by these techniques are not substantially different. These results suggest the transgenic rice and rice from MAB breeding that were compared in the current study are substantially equivalent at the molecular and biological levels. The data and results can be used to study chemical and nutritional equivalence of rice generated by transgenesis and MAB breeding.

## Methods

### Resistance analysis of rice varieties

Both D62B and MH86 are *indica* rice and widely used as parental lines of hybrid rice in China. The transgenic line DXT with *Xa21* was developed from D62B in our previous study [22]. The MAB line DXB with *Xa21* was developed by backcrossing to D62B in the current study. When the rice grew to the high tillering stage, five to seven fully expanded leaves were inoculated with *Xoo* using the leaf-clipping method [49]. The cultures were grown on PSA (Potato-Sugar-Agar) medium (potato, 300 g/L; Ca(NO_3_)_2_•4H_2_O, 0.5 g/L; Na_2_HPO_4_•12H_2_O, 2.0 g/L; sugar, 15 g/L; agar 15 g/L) at 28°C for 3 days. Inoculums were prepared by suspending the bacterial cells in sterile water and adjusting the concentration to about 10^9^ cells per milliliter. Phenotype scoring was carried out at 15 days post innoculation (dpi).

### Molecular analysis of transgenic and MAB breeding plants

Genomic DNA was isolated from fresh rice leaf tissue using the cetyltrimethylammonium bromide protocol. Transgenic plants were validated by polymerase chain reaction (PCR) and Southern blotting. The primers which were used for molecular analysis of transgenic plants were described detailedly in Gao et al. (2011). MAB breeding plants were validated by PCR using the same primers as transgenic plants.

### Quantitative PCR

DNaseI-treated RNA was used for fist strand cDNA synthesis using M-MLV reverse transcriptase (Promega) and oligo(dT)_15_ according to the manufacture’s protocols. Specific pairs of primers for SYBR-green detection and quantification of selected DEGs were designed using Primer Express software follwing the primer design guidance. The primer sequences are listed in Additional file 1: Table S5. The endogenous actin gene was run in parallel as control PCR reaction and the untransfromed receptor line was used as the calibrator to normlize the relative expression levels of the target DEG. Triplicate samples for each tested line were prepared for real-time PCR assays. The 2^-∆∆CT^ method was used to calculate relative changes of gene expression [50]. PCR reactions were performed using the TranStart Green qPCR SupMix reagent (TransGen Biotech, Inc.) on BioRad CFX96 PCR system.

### Illumina sequencing

Sequencing and preliminary data acquisition were finished by Beijing Genomics Institute. The experimental process includes sample preparation and sequencing. The main reagents and supplies used included Illumina Gene Expression Sample Prep Kit and Solexa Sequencing Chip (flowcell), and the main instruments used included Illumina Cluster Station and Illumina HiSeq™ 2000 Systerm. Ten leaves randomly selected from ten individual plants of each tested line were harvested and pooled together for RNA extraction. Total RNA was isolated using Trizol reagent (Invitrogen Corporation, Carlsbad, CA, USA) following the manufacture’s protocols and purified using an RNeasy Plant Kit (Qiagen, Hilden, Germany). The integrity and purity of RNA samples were determined by gel electrophoresis and OD 260/280 nm absorption ratios and the RNA concentration was quantified using a NanoDrop ND-1000 spectrophotometer (NanoDrop Technology, Wilmington, DE, USA).

### Processing of sequencing data

Raw sequencing data in image were transformed by base calling into raw reads. Raw reads were then transformed into clean reads after removal of such reads as 3' adaptor sequences, empty reads, low quality reads, reads which were too long or too short, and reads with only one copy (probably due to sequencing error). Clean reads that were mapped to reference sequences from multiple genes were removed. The remaining clean reads were designated as unambiguous clean or qualified reads.

### Gene expression and differentially expressed genes

The number of qualified reads for each gene was tallied and normalized to TPM (number of transcripts per million qualified reads), which was then used as the digital gene expression abundance of the gene. Genes that were differentially expressed, referred to as DEGs, across two plants were identified using the criterion of at least 2-fold change to digital expression abundance and with a cut-off of False Discovery Rate (FDR) no greater than 0.001.

### Cluster analysis

All DEGs in DXT, DXB and MH86, with respect to D62B, were aggregated. The log_10_-transformed TPM values of the genes in the combined set of DEGs were used to cluster the DEGs by hierarchical clustering with euclidean distance and average linkage. The clustering was done and the result was plotted using Multi Experiment Viewer (MeV) [51].

### Gene ontology and KEGG pathway analyses

GO functional category analysis was performed separately on DEGs and genes exclusively expressed in a specific plant, e.g., DXT or DXB. This analysis was done by mapping the set of genes of interest (DEGs or plant-specific genes) to the terms in plant GOSlim Ontologies from the Rice Genome Annotation Project (http://rice.plantbiology.msu.edu/annotation_pseudo_goslim.shtml). WEGO (Web Gene Ontology Annotation Plot [52]) tool was used to plot GO annotation results. For pathway enrichment analysis, all DEGs involved in a comparison were mapped to the terms in the KEGG database to identify significantly enriched KEGG terms. The Kobast 2.0 [53] tool for pathway enrichment analysis was used with a cutoff of *p* value no greater than 0.05.

### Availability of supporting data

The gene expression profiling data on the four rice plants from Illumina HiSeq™ 2000 has been deposited into NCBI/GEO database under the accession number SRA061839.

## Competing interests

The authors declare that they have no competing interests.

## Authors’ contributions

LG carried out the molecular analysis and field score of rice varieties, analyzed the results and contributed to the drafting of the manuscript; YC and GL participated in data analysis; ZX performed qPCR experiments; GJ analyzed the leaf blight resistance of transgenic and MAB rice; WZhang conceived the research topic, analyzed the results and wrote the paper; WZhai conceived the study, designed the experiments and coordinated the research. All authors read and approved the final manuscript.

## Supplementary Material

Additional file 1: Table S1 Differentially expressed genes in transgenic rice line DXT, MAB breeding line DXB and a rice variety MH86 with respect to the expression in D62B. **Table S2.** Pathways enriched in the differentially expressed genes in transgenic rice line DXT with respect to the expression in the parental line D62B. **Table S3.** Pathways enriched in the differentially expressed genes in MAB breeding line DXB with respect to the expression in the parental line D62B. **Table S4.** Pathways enriched in the differentially expressed genes in rice variety MH86 with respect to the expression in D62B. **Table S5.** Primers used in qRT-PCR analysis of ten differentially expressed genes. Click here for file

Additional file 2: Figure S1 Real-time PCR analysis of 10 differentially expressed genes. Expression patterns of LOC_Os10g41838, LOC_Os08g20500, LOC_Os11g39320, LOC_Os09g26560, LOC_Os11g39190, LOC_Os10g30970, LOC_Os09g36420 and LOC_Os09g19229 are consistent with digital gene expression abundance from deep sequencing. Triplicate samples for each tested lines were used for real-time PCR assays. Actin gene was used as internal controls and the recipient D62B was used as the calibrator. The 2^-∆∆CT^ method was used to calculate relative changes in gene expression. The vertical axes represent the relative expression levels of target genes with respect to the control of actin gene. DXT: transgenic line with *Xa21*; DXB: MAB line with *Xa21*; D62B: the common parental line of DXT and DXB. Click here for file
