# Supplementary figures and images for: Do transgenesis and marker-assisted backcross breeding produce substantially equivalent plants? - A comparative study of transgenic and backcross rice carrying bacterial blight resistant gene Xa21
Source: BMC Genomics. 2013 Oct 29;14:738. doi: 10.1186/1471-2164-14-738 (PMC4007521; doi:10.1186/1471-2164-14-738)

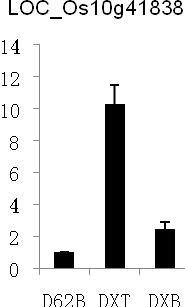

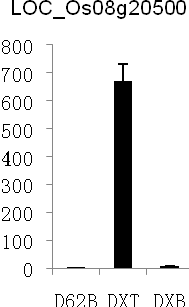

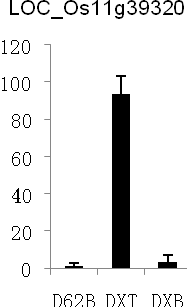

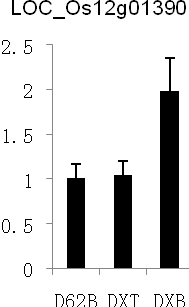

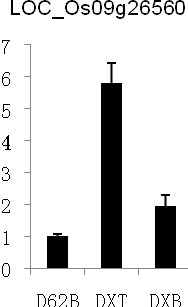


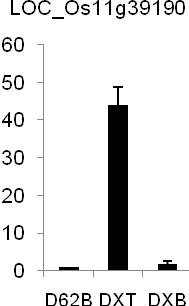

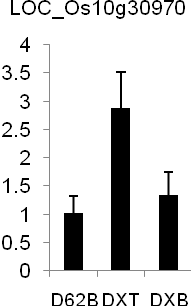

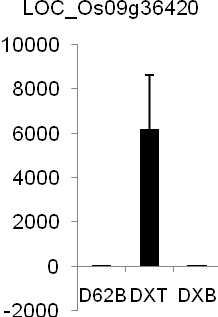

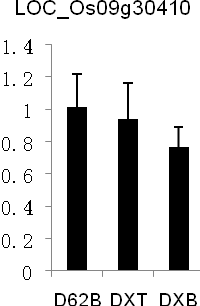

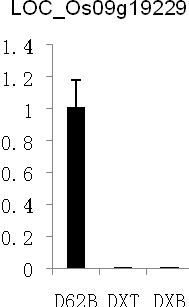

Supplement: Additional file 2: Figure S1 — Real-time PCR analysis of 10 differentially expressed genes. Expression patterns of LOC_Os10g41838, LOC_Os08g20500, LOC_Os11g39320, LOC_Os09g26560, LOC_Os11g39190, LOC_Os10g30970, LOC_Os09g36420 and LOC_Os09g19229 are consistent with digital gene expression abundance from deep sequencing. Triplicate samples for each tested lines were used for real-time PCR assays. Actin gene was used as internal controls and the recipient D62B was used as the calibrator. The 2-∆∆CT method was used to calculate relative changes in gene expression. The vertical axes represent the relative expression levels of target genes with respect to the control of actin gene. DXT: transgenic line with Xa21; DXB: MAB line with Xa21; D62B: the common parental line of DXT and DXB. [file 1471-2164-14-738-S2.doc]
